# Prolonged Calorie Restriction Downregulates Skeletal Muscle mTORC1 Signaling Independent of Dietary Protein Intake and Associated microRNA Expression

**DOI:** 10.3389/fphys.2016.00445

**Published:** 2016-10-05

**Authors:** Lee M. Margolis, Donato A. Rivas, Maria Berrone, Yassine Ezzyat, Andrew J. Young, James P. McClung, Roger A. Fielding, Stefan M. Pasiakos

**Affiliations:** ^1^Nutrition, Exercise, Physiology, and Sarcopenia Laboratory, U.S. Department of Agriculture Jean Mayer Human Nutrition Research Center on Aging, Tufts UniversityBoston, MA, USA; ^2^Military Nutrition Division, US Army Research Institute of Environmental MedicineNatick, MA, USA

**Keywords:** muscle protein content, energy deficit, rpS6, miR-99, miR-100

## Abstract

Short-term (5–10 days) calorie restriction (CR) downregulates muscle protein synthesis, with consumption of a high protein-based diet attenuating this decline. Benefit of increase protein intake is believed to be due to maintenance of amino acid-mediated anabolic signaling through the mechanistic target of rapamycin complex 1 (mTORC1), however, there is limited evidence to support this contention. The purpose of this investigation was to determine the effects of prolonged CR and high protein diets on skeletal muscle mTORC1 signaling and expression of associated microRNA (miR). Twelve-week old male Sprague Dawley rats consumed *ad libitum* (AL) or calorie restricted (CR; 40%) adequate (10%, AIN-93M) or high (32%) protein milk-based diets for 16 weeks. Body composition was determined using dual energy X-ray absorptiometry and muscle protein content was calculated from muscle homogenate protein concentrations expressed relative to fat-free mass to estimate protein content. Western blot and RT-qPCR were used to determine mTORC1 signaling and mRNA and miR expression in fasted mixed gastrocnemius. Independent of dietary protein intake, muscle protein content was 38% lower (*P* < 0.05) in CR compared to AL. Phosphorylation and total Akt, mTOR, rpS6, and p70S6K were lower (*P* < 0.05) in CR vs. AL, and total rpS6 was associated with muscle protein content (*r* = 0.64, *r*^2^ = 0.36). Skeletal muscle miR expression was not altered by either energy or protein intake. This study provides evidence that chronic CR attenuates muscle protein content by downregulating mTORC1 signaling. This response is independent of skeletal muscle miR and dietary protein.

## Introduction

Calorie restriction (CR) is a strategy to lose body fat and reduce total body mass. However, skeletal muscle is also lost during CR, which may compromise successful weight management and negatively impact physical function. The loss of skeletal muscle may be due in part to the effects of CR on muscle protein synthesis (MPS; Pasiakos et al., 2015). Recent studies have demonstrated that CR, particularly short-term CR (≤ 21 days), downregulates fasting MPS and blunts anabolic sensitivity to a protein-containing meal (Pasiakos et al., 2010; Areta et al., 2014; Hector et al., 2015; Murphy et al., 2015). Increasing dietary protein intake above the recommended dietary allowance (RDA; 0.8 g·kg^−1^·d^−1^) may attenuate declines in MPS, preserve anabolic sensitivity to a protein-containing meal, and spare skeletal muscle mass during short-term CR (Pasiakos et al., 2013; Areta et al., 2014). The anabolic sparing effects of high protein diets during short-term CR are likely mediated by the mechanistic target of rapamycin complex 1 (mTORC1; Proud, 2007; Drummond et al., 2009; Pasiakos, 2012), although evidence to support this contention is limited (Pasiakos et al., 2010; Miller et al., 2013; Areta et al., 2014). Despite changes in MPS, altered mTOC1 signaling under fasting conditions has not been shown during short-term CR with adequate or high protein diets. Whether prolonged CR and dietary protein level affect mTORC1 signaling, and result in modifications in fat-free mass has not been determined.

Recently, microRNA (miR) have been identified as novel regulators of skeletal muscle mass (Baek et al., 2008; Selbach et al., 2008; Rivas et al., 2014), with specific miR serving as negative regulators of hypertrophy through mRNA degradation or repression of translation for mTORC1 associated signaling proteins (Pillai et al., 2004; Kong et al., 2008). In acute cell culture models, overexpressing members of the miR-99/100 family inhibits gene expression and protein translation of mTORC1 signaling proteins, downregulating the activity of the pathway and ultimately cellular proliferation (Jin et al., 2013; Wei et al., 2013; Jia et al., 2014). In human muscle, diminished miR-99 and miR-100 expression predict the anabolic response to a resistance exercise bout, with reduced expression of these miR upregulating mTORC1 signaling (Zacharewicz et al., 2014). While acute alterations in miR expression have been associated with skeletal muscle mTORC1 signaling in some studies, no investigations have assessed whether prolonged CR and dietary protein level augment miR expression, subsequent translational modifications in mTORC1 signaling, and whether miR are associated with skeletal muscle mass in response to underfeeding.

The objectives of this study were to: (1) determine the effects of prolonged CR on mTORC1 signaling, (2) determine the extent to which high protein diets modulate the mTORC1 response, (3) assess whether CR and dietary protein-mediated changes in mTORC1 signaling influence skeletal muscle, and (4) explore the potential regulatory role of miR. We hypothesized that prolonged CR would downregulate mTORC1 signaling resulting in lower fat-free mass after CR. We expected that consuming a high protein diet would prevent declines in mTORC1 signaling and spare fat-free mass. We hypothesized that CR and dietary protein-mediated changes in mTORC1 signaling would result from translational modifications secondary to altered expression of associated miR.

## Experimental design

Twelve-week-old male Sprague Dawley rats (*n* = 40; Charles River Laboratories) were randomized by body mass to one of four diet groups consuming *ad libitum* (AL) or calorie restricted (CR; 40% less feed compared to AL) with standard (10%) or high (32%) protein (PRO) diets for 16-weeks. This investigation was part of a larger study designed to assess the influence of protein source (soy vs. milk-based protein) on intestinal calcium absorption and bone (Gaffney-Stomberg et al., 2014). Only the rats consuming the milk-based protein diet were analyzed in the current study due to the amino acid content of the standard and high milk-based protein diets (Pasiakos and McClung, 2011). We wanted to test our hypothesis that dietary protein level modulates mTORC1 during sustained CR without protein quality (i.e., amino acid content) being a potential confounder. All study procedures were approved by the US Army Research Institute of Environmental Medicine Animal Care and Use Committee.

### Diet

Purified study diets based on AIN-93 (Dyets, Inc., Bethlehem, PA) were modified to provide 10 and 32% protein to align with the lower and upper end of the current acceptable macronutrient distribution range (milk protein concentrate, Idaho Milk Products, Jerome, ID) (Gaffney-Stomberg et al., 2014). Milk protein concentrate was chosen because of its amino acid composition, digestion and absorption kinetics, and its effects on muscle intracellular signaling (Pasiakos, 2015). Chemical analysis of diets was performed to ensure nutrient content (Covance Laboratories, Dedham, MA; Table 1). The amount of feed provided to the CR rats was initially determined by averaging daily intake during the 14-d acclimation phase. Feed intake for CR fed rats was 16 ± 2 g·d^−1^ whereas the AL rats consumed 26 ± 3 g·d^−1^. Feed intake for the AL rats was assessed every 2 days so that adjustments could be made to ensure the CR rats maintained a 40% energy deficit.

**Table 1:** **Energy and macronutrient composition of experimental diets^a^**.

	**AL 10% PRO**	**AL 32% PRO**	**CR 10% PRO**	**CR 32% PRO**
Energy (kcal·d^−1^)	95.1	96.7	57.1	58.0
Protein (g·d^−1^)	2.4	8.2	1.4	4.9
BCAA (mg·d^−1^)^b^				
Leucine	242.0	900.0	145.2	540.0
Isoleucine	127.5	482.5	76.5	289.5
Valine	145.0	550.0	87.0	330.0
Carbohydrate (g·d^−1^)	17.1	10.9	10.3	6.7
Fat (g·d^−1^)	1.9	2.3	1.1	1.4

a *Diets formulated based on AIN-93 with ad libitum fed rats consuming 25 g·d^-1^ and Calorie Restricted fed rats consuming 15 g·d^-1^*.

b *BCAA, branched-chain amino acid*.

### Body mass and composition

Body mass was determined twice a week using a calibrated electronic scale (Ohaus, East Lyme, CT). Dual Energy X-ray Absorptiometry (DXA; Lunar iDXA, GE Lunar Corp., Madison, WI, USA) was used to assess body composition after the16 week feeding intervention under anesthesia [1 mL·kg^−1^ mixture of 40 mg·kg^−1^ ketamine (Ketaset; Fort Dodge Animal Health, Fort Dodge, IA), 10 mg·kg^−1^ xylazine (Xyla-Ject; Phoenix Scientific, Inc., St. Joseph, MO), and 1.0 mg·kg^−1^ acepromazine (Boehringer Ingelheim, St. Joseph, MO)]. Small animal software (enCore Version 11.40.004, 2007; GE Lunar Corp.) was used to determine body composition.

### Muscle protein content

Whole gastrocnemius samples were homogenized under liquid nitrogen using a mortar and pestle. Sample was then placed ice-cold homogenization buffer (1:10 wt/vol) containing 50 mM Tris–HCl (pH 7.5), 5 mM Na-pyrophosphate, 50 mM NaF, 1 mM EDTA, 1 mM EGTA, 10% glycerol (v/v), 1% Triton-X, 1 mM DTT, 1 mM benz-amidine, 1 mM PMSF, 10 μg mL^−1^ trypsin inhibitor and 2 μg mL^−1^ aprotinin. Protein concentration of the homogenate was then determined using 660 nm Protein Assay (ThermoFisher Scientific, Waltham, MA, USA). Homogenate total protein concentration was expressed relative to DXA derived fat-free mass to provide an estimate of muscle protein content (Thomson and Gordon, 2005, 2006). The muscle protein content measure accounts for potential differences in water weight (Ianuzzo and Chen, 1979; Thomson and Gordon, 2006) and provides an assessment of protein content, allowing for an estimate of muscle growth, not whole-body fat-free mass, independent of water weight. Muscle protein content was calculated as:

$$\begin{array}{l} \text{Muscle protein content}=\text{protein concentration }(\text{mg }\cdot \;{\text{ml}}^{-\text{1}})\\ \;\times \;(\text{homogenate vol}(\text{ml})\\ \;/\text{muscle wt}(\text{mg}))\times \text{ FFM}(\text{g}). \end{array}$$

### Intracellular signaling

Phosphorylation status and total protein expression of molecular markers associated with mTORC1 signaling were determined using Western blot. Homogenates used for muscle protein content estimations were centrifuged for 15 min at 10,000 × g at 4°C, the supernatant (lysate) was collected and protein concentration analyzed (ThermoFisher). Muscle lysates were solubilized in Laemmli buffer, with equal amounts of total protein (10 μg) and separated by SDS-PAGE using precast Tris·HCl gels (Bio-Rad Laboratories, Hercules, CA, USA). Proteins were transferred to polyvinylidene fluoride membranes and exposed to commercially available primary antibodies specific to Akt, p-Akt^Ser473^, mTOR, p-mTOR^Ser2448^, p70S6K, p-p70S6K^Thr389^, rpS6, p-rpS6^Ser235/236^, AMPKα (Cell Signaling Technology, Danvers, MA, USA), and PGC-1α (Santa Cruz, Dallas, TX USA) at 4°C overnight. Labeling was performed using secondary antibody (anti-rabbit IgG conjugate with horseradish peroxidase; Cell Signaling Technology), and chemiluminescent reagent was applied (Super Signal, West Pico Kit; Pierce Biotechnology, Rockford, IL, USA). Blots were quantified using a phosphoimager (ChemiDoc XRS; Bio-Rad) and Image Lab software (Bio-Rad). To confirm equal protein loading per well glyceraldehyde 3-phosphate dehydrogenase (GAPDH) was used for mTORC1 associated proteins and heat shock protein 90 (HSP90) was used for AMPKα and PGC-1α. Phosphorylation and total protein were normalized to GAPDH or HSP90 and the ratio of phosphorylation-to-total protein was determined. All data are presented as fold change relative to AL 10% PRO.

### mRNA and miR expression

mRNA expression of genes associated with intracellular regulation of muscle mass, including mTORC1 amino acid sensing proteins (Lars and Map4k3; TaqMan®), amino acid transporters (Slc38a2 and Slc7a5 TaqMan®), and energy utilization (Sirt1, Ppargc1a, Tfam, Ppara, Pparg; Bio-Rad), were determined using commercial available primers and in mixed gastrocnemius samples. miR that regulate targets in the mTORC1 pathway (hsa-miR-16-5p, hsa-miR-26b-5p, hsa-miR-99a-5p, hsa-miR-100-5p, hsa-miR-128a-3p, hsa-miR-133a-3p, hsa-miR-199a-3p, hsa-miR-221-3p) were analyzed using TaqMan® microRNA Assays (Applied Biosystems, Foster City, CA, USA). The roles of the mRNA and miR assessed are provided in Supplemental Table 1.

Total RNA was isolated in 20 mg muscle samples using a mirVana™ miRNA isolation kit (Invitrogen, Carlsbad, CA, USA); RNA quantity and quality were assessed using a Nanodrop ND-1000 spectrophotometer (Nanodrop, Wilmington, DE, USA). Equal amounts of total RNA were synthesized into cDNA (iScript™ Advanced cDNA Synthesis Kit; Bio-Rad). miR were reverse-transcribed using the TaqMan® microRNA RT kit (Applied Biosystems) with the nine miR-specific stem-loop reverse transcript (RT) primers pooled in 1X-Tris-EDTA (TE) buffer for a final dilution of 0.05X for each miR RT primer. The RT primer pool (6 μl) was added to the RT reaction mix (0.3 μl 100 mM dNTP, 3 μl enzyme, 1.5 μl 10 × RT buffer, 0.19 μl RNase inhibitor) and 250 ng of total RNA (Le Carre et al., 2014). For both mRNA and miR, reverse transcription was conducted using a T100™ Thermal Cycler (Bio-Rad). RT-qPCR amplifications were conducted using CFX96 Touch™ Real-Time PCR Detection System (Bio-Rad). Samples were run in 20 μl reactions in triplicates, using iTaq™ Universal SYBR® Green Supermix (Bio-Rad) of mRNA, and TaqMan® Univsersal PCR MasterMix (2X), no UNG (Applied Biosystems) for miR. All mRNA and miR were normalized to the geometric mean of U6 and B2M. Fold changes for mRNA and miR were calculated using the ΔΔ cycle threshold (ΔΔC_*T*_) method (Pfaffl, 2001) and expressed relative to AL 10% PRO.

### Citrate synthase activity assay

Citrate synthase activity was assessed using whole cell lysate from homogenized mixed gastrocnemius samples as described above. Enzyme activity was determined using a colorimetric assay analyzed on an ELx808 Absorbance Reader (BioTek®, Winooski, VT, USA), by combining 10 μl of diluted (1:10; 0.1 M Tris HCl pH 8.1) sample to 150 μl of reaction master mix (1 mL DNTB, 3 mg Acetyl CoA, and 8 mL 0.1 M Tris HCl pH 8.1). The reaction was initiated when 10 μl of 10 mM oxaloacetate was added to each well. Samples were read at 412 nm. Data were normalized to protein content.

### Plasma amino acid concentrations

Plasma amino acid concentrations were determined from blood collected by intra-cardiac puncture. Samples were tested in duplicate using HPLC and o-phthaldialdehyde post-column derivatization (Agilent 1100 Series HPLC; Agilent Technologies). Amino acid concentrations were used to determine the influence of dietary protein and energy status on mTORC1 signaling.

### Statistical analysis

A repeated measures ANOVA was used to determine influence of time (Baseline vs. Week 16), energy status (AL vs. CR) and protein intake (10 vs. 32%) on body mass and composition. A univariate ANOVA was conducted to determine the influence of energy status and protein intake on muscle protein content, Western blots, mRNA and miR expression, and serum BCAA. Bonferroni adjustments for multiple comparisons were performed if significant interactions (energy-by-protein) were observed. Spearman's rank correlation coefficients were used to determine the relationship between muscle protein content, as determined by protein muscle content, and total Akt, mTOR, p70S6K, and rpS6. All mRNA and miR data were log transformed (Log_2_) for statistical analysis, but presented as original values (median ± SEM). All other data presented as mean ± SEM. The α level for significances was set at *P* < 0.05. Data were analyzed using IBM SPSS Statistics for Windows Version 22.0 (IBM Corp. Armonk, NY).

## Results

### Body mass, composition and muscle protein content

**Figure 1:**
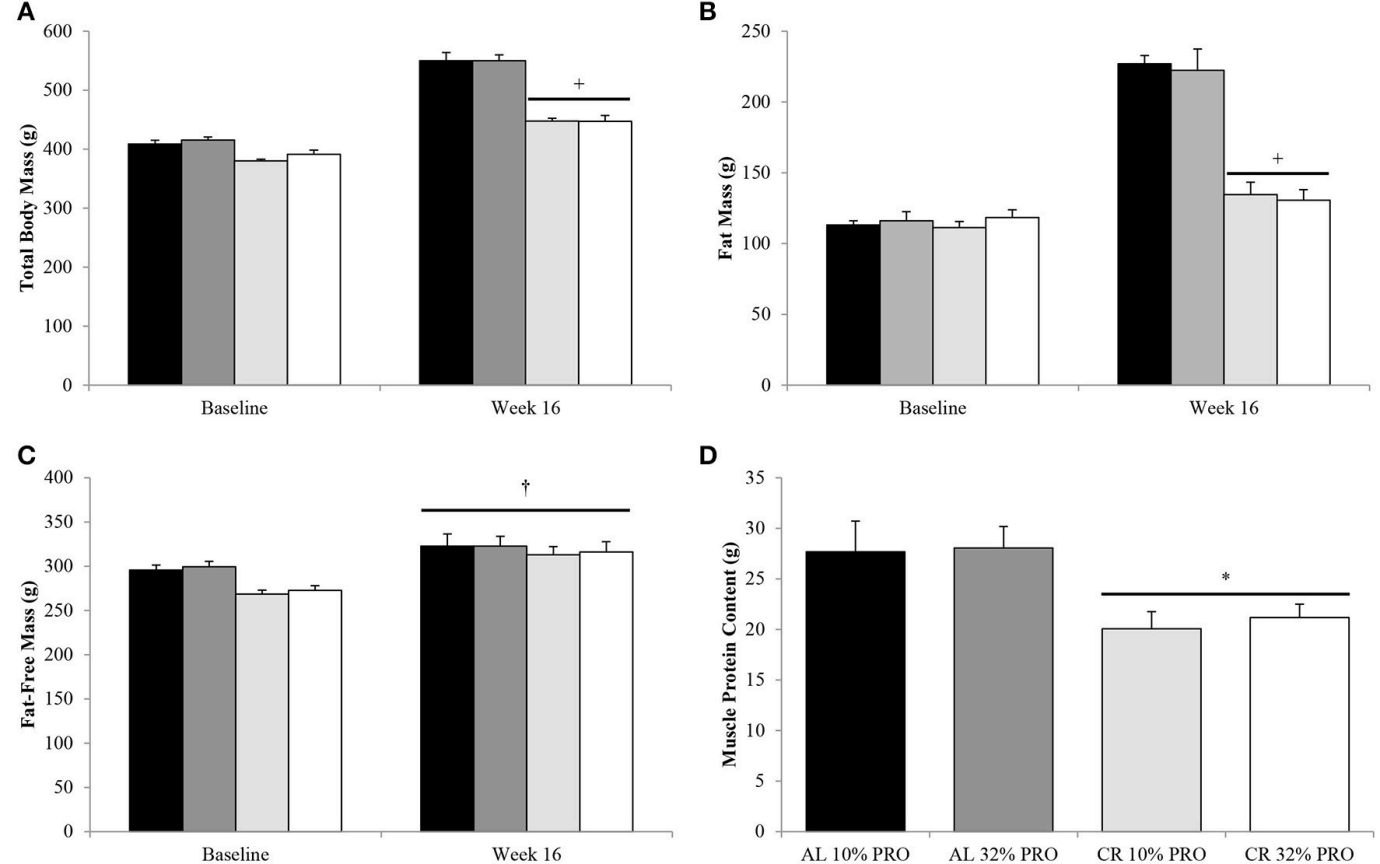
**Mean (± SEM) [*n* = 10 (AL 10% PRO; 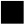), *n* = 10 (AL 32% PRO; 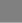), *n* = 10 (CR 10% PRO; 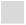), *n* = 10 (CR 32% PRO; □)] body mass (A), fat mass (B), fat-free mass (C), and muscle protein content (D)**. Data analyzed using a univariate ANOVA with Bonferroni correction to determine main effects of energy (AL vs. CR), protein (10 vs. 32%) and energy-by-time interactions. ^†^Week 16 different from Baseline; *P* < 0.05. ^+^Time-by-energy interaction, CR different from AL at week 16; *P* < 0.05. ^*^CR different from AL; *P* < 0.05.

Following the 16-week feeding intervention a time-by-energy interaction was observed, with change in total body and fat mass were lower (*P* < 0.05) for CR compared to AL, regardless of protein intake (Figures 1A,B). Fat-free mass increased (*P* < 0.05) from baseline to week 16 in all rats, with no effect of energy or protein intake (Figure 1C). Though fat-free mass was statistically similar between CR and AL at the conclusion of the 16-week feeding intervention, muscle protein content was 38% lower (7 ± 2 g; *P* < 0.05) in CR than AL (Figure 1D).

### Anabolic intracellular signaling

**Figure 2:**
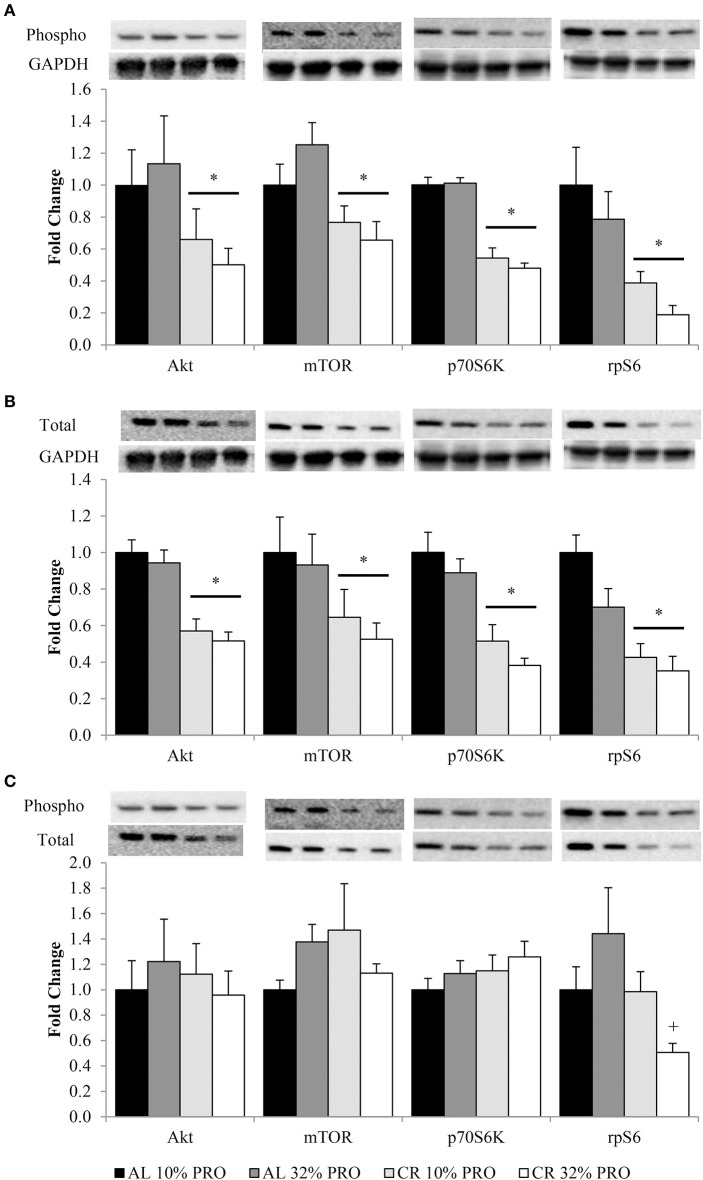
**Mean (± SEM) [*n* = 10 (AL 10% PRO), *n* = 10 (AL 32% PRO), *n* = 10 (CR 10% PRO), *n* = 10 (CR 32% PRO)] phosphorylation (A) and totals (B) relative to GAPDH, and phosphorylation relative to total (C)**. Data analyzed using a univariate ANOVA with Bonferroni correction to determine main effects of energy (AL vs. CR), protein (10 vs. 32%) and energy-by-protein interactions. ^*^CR body mass different from AL; *P* < 0.05.+Energy-by-protein interaction, CR 32% PRO different than AL 32% PRO; *P* < 0.05.

**Figure 3:**
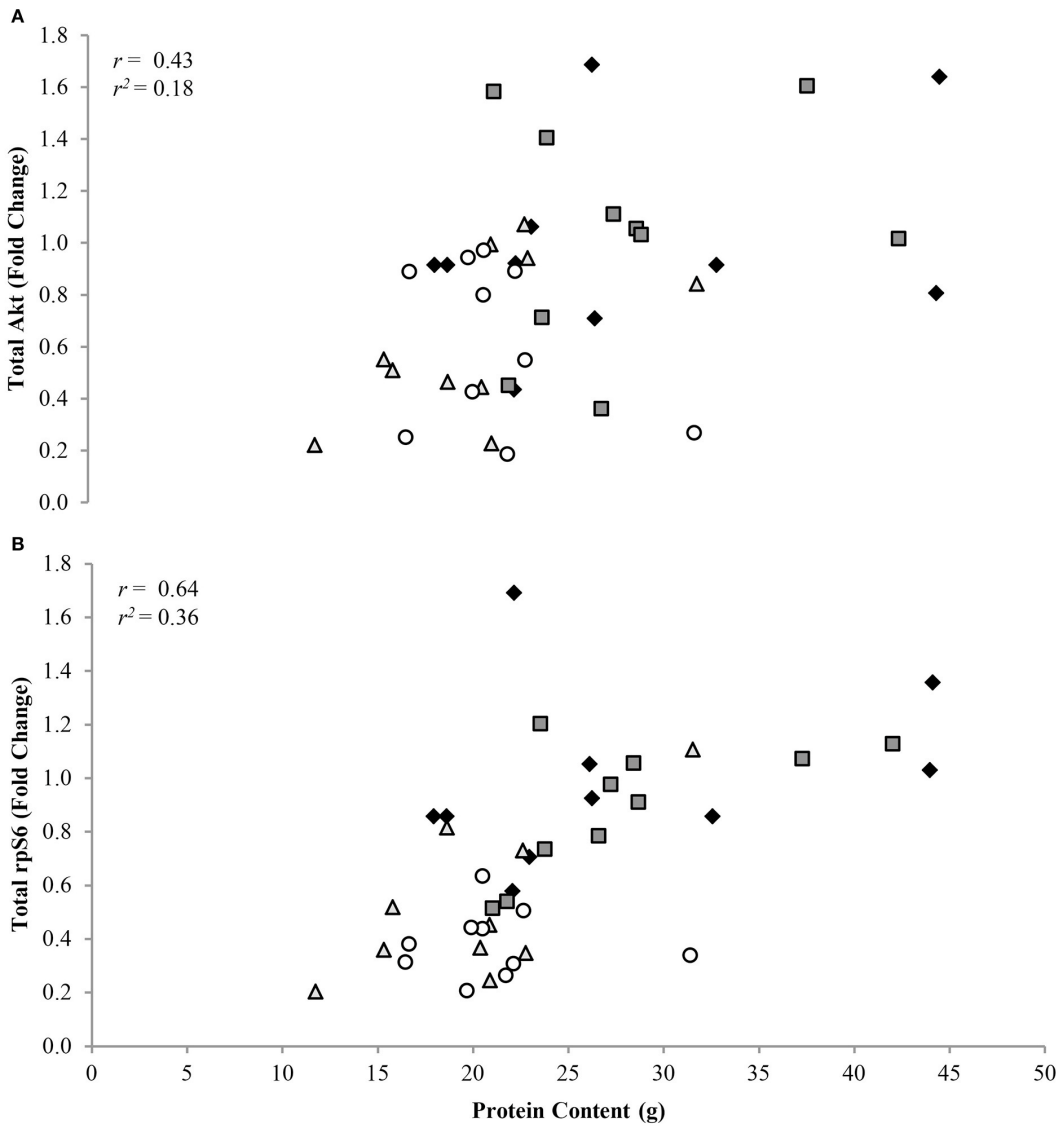
**Association of total Akt (A) and rpS6 (B) to muscle protein content [*n* = 10 (AL 10% PRO ♦), *n* = 10 (AL 32% PRO 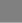), *n* = 10 (CR 10% PRO 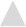), *n* = 10 (CR 32% PRO ◦)]**. Data analyzed using a spearman rho correlation coefficient. Significant associations were determined as *P* < 0.05.

Phosphorylation status of Akt^Ser473^, mTOR^Ser2448^, p70S6K^Thr389^, and rpS6^Ser235/236^ were (*P* < 0.05) 1.72 ± 0.11, 1.41 ± 0.08, 1.95 ± 0.05, and 3.47 ± 0.05 fold lower, respectively, in CR compared to AL rats, regardless of protein intake (Figure 2A). Total protein of Akt, mTOR, p70S6K, and rpS6 were 1.85 ± 0.04, 1.71 ± 0.08, 2.23 ± 0.05, and 2.57 ± 0.05 fold lower, respectively, in CR compared to AL rats, with no effect of dietary protein intake (Figure 2B). Energy or dietary protein did not impact the ratio of phosphorylation-to-total protein, except for an energy-by-protein interaction for rpS6, where CR 32% PRO was 1.98 ± 0.22 fold lower than AL 10% PRO (Figure 2C). Akt (*r* = 0.43, *r*^2^ = 0.18; Figure 3A) and rpS6 (*r* = 0.64, *r*^2^ = 0.36; Figure 3B) were positively associated (*P* < 0.05) with protein content. No other associations were observed.

### mRNA and miR expression

**Figure 4:**
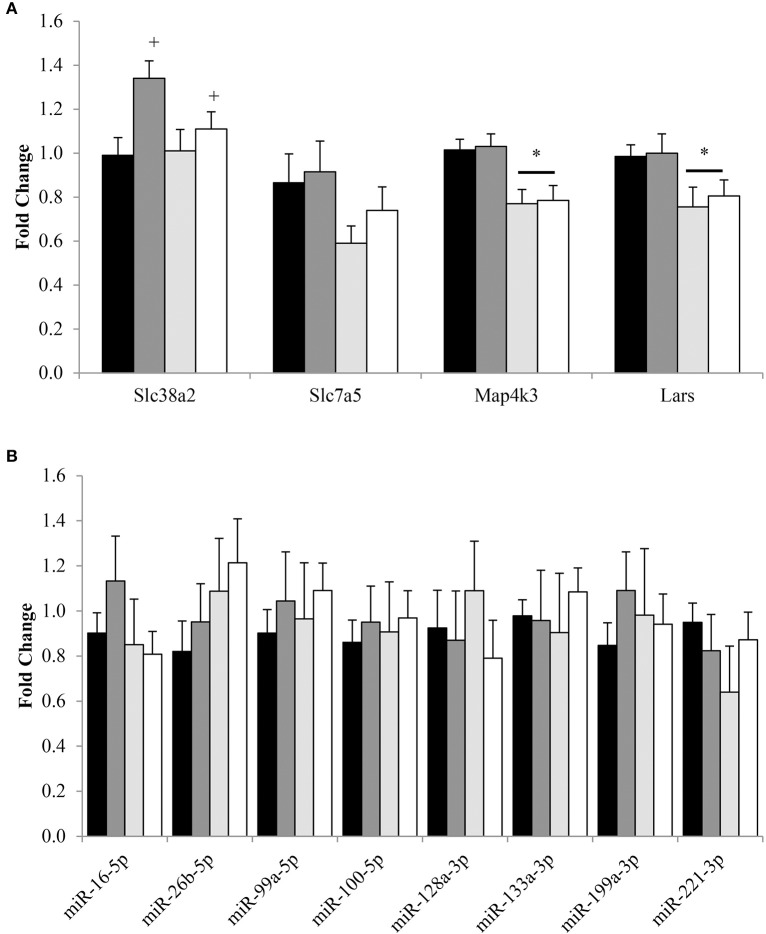
**Median (± SEM) [*n* = 10 (AL 10% PRO; 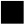), *n* = 10 (AL 32% PRO; 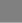), *n* = 10 (CR 10% PRO; 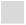), *n* = 10 (CR 32% PRO; □)] mRNA (A) and miR (B) expression**. Data analyzed using a univariate ANOVA with Bonferroni correction to determine main effects of energy (AL vs. CR), protein (10 vs. 32%) and energy-by-protein interactions. ^*^CR body mass different from AL; *P* < 0.05. ^+^32% PRO different than 10%; *P* < 0.05.

Energy and protein intake altered the expression of genes associated with amino acid sensing and transport upstream of mTORC1 (Figure 4A). Regardless of energy intake, amino acid transporter Slc38a2 expression was 1.19 ± 0.08 fold higher (*P* < 0.05) for 32% PRO vs. 10% PRO. No effect of energy or protein intake was observed for Slc7a5. Expression of amino acid sensing Map4k3 and Lars were 1.28 ± 0.06 and 1.28 ± 0.08 fold lower (*P* < 0.05) for CR than AL, independent of dietary protein. No effects of energy or protein intake were observed in the expression of any miR (Figure 4B).

### Energy utilization

**Figure 5:**
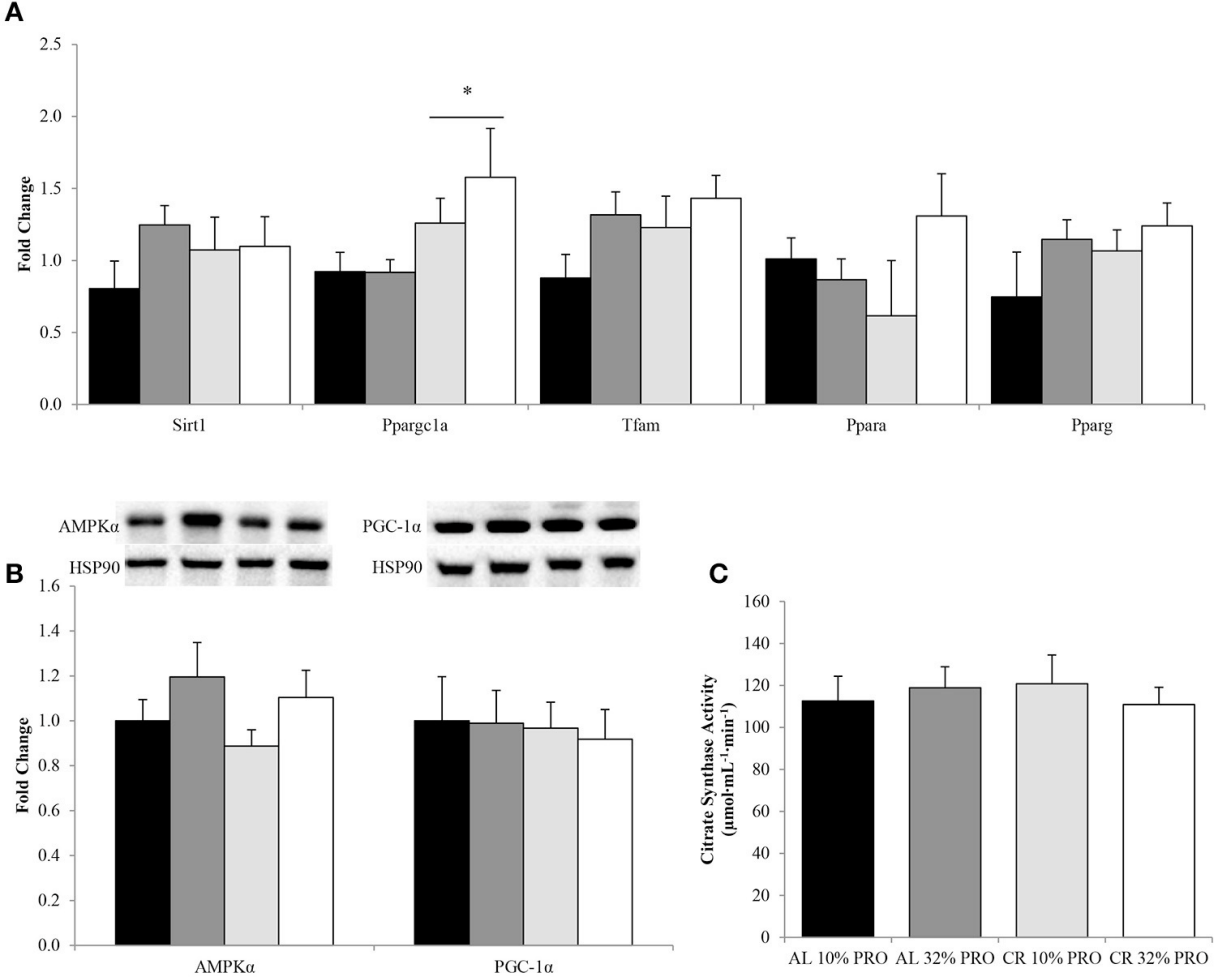
**Median (± SEM) [*n* = 10 (AL 10% PRO; 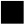), *n* = 10 (AL 32% PRO; 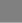), *n* = 10 (CR 10% PRO; 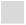), *n* = 10 (CR 32% PRO; □)] mRNA (A) and mean (± SEM) total AMPKα and PGC-1α (B) expression and citrate synthase activity (C)**. Data analyzed using a univariate ANOVA with Bonferroni correction to determine main effects of energy (AL vs. CR), protein (10 vs. 32%) and energy-by-protein interactions. ^*^CR different from AL; *P* < 0.05.

Regardless of protein intake, Ppargc1a expression was 1.74 ± 0.20 fold higher (*P* < 0.05) in CR compared to AL (Figure 5A). No effect of energy or protein was observed for gene expression of Sirt1, Tfam, Ppara, and Pparg. Total protein for master regulators of energy utilization, AMPKα and PGC-1α, were similar between AL and CR (Figure 5B). Additionally, citrate synthase activity was also similar between CR and AL (Figure 5C). No effect of protein was observed for AMPKα or PGC-1α protein expression and citrate synthase activity.

### Plasma branched-chain amino acid profile

A main effect of energy was observed for plasma branched-chain amino acids; leucine, isoleucine and valine concentrations were 25.9 ± 5.0, 19.2 ± 3.1, and 27.3 ± 5.5 μmol·L^−1^ lower (*P* < 0.05) in CR than AL (Table 2). A main effect of protein was also observed for branched-chain amino acids; leucine, isoleucine, and valine concentrations were 12.5 ± 5.0, 6.6 ± 3.1, and 19.6 ± 5.5 μmol·L^−1^ higher (*P* < 0.05) in rats consuming 32% PRO vs. 10% PRO. No energy-by-protein interactions were observed.

**Table 2:** **Plasma branched-chain amino acids profiles following 16-week feeding intervention**.

**Amino acids (μmol·L^−1^)**	**AL 10% PRO**	**AL 32% PRO**	**CR 10% PRO**	**CR 32% PRO**	***P*****-value**		
					**Energy**	**Protein**	**Energy x Protein**
BCAA^a^	442.5 ± 7.4	472.6 ± 17.8	361.5 ± 14.9	408.8 ± 10.7	<0.01	<0.01	0.52
Leucine	154.0 ± 3.8	166.7 ± 6.6	128.3 ± 5.5	140.6 ± 3.9	<0.01	0.02	0.97
Isoleucine	93.5 ± 1.8	96.9 ± 3.9	71.1 ± 3.8	80.9 ± 2.5	<0.01	0.04	0.31
Valine	195.0 ± 3.0	209.0 ± 7.5	162.1 ± 5.8	187.3 ± 4.9	<0.01	<0.01	0.32

*Mean ± SEM; Univariate ANOVA*.

a *BCAA, branched-chain amino acids*.

## Discussion

The primary findings from this investigation were that prolonged CR (16-weeks; 40% total energy requirements) led to downregulation of fasting mTORC1 signaling activity and inhibition of protein translation and phosphorylation (i.e., activity) of Akt, mTOR, p70S6K, and rpS6 under fasted conditions. The systematic downregulation of mTORC1 associated protein expression and activity, particularly the decline rpS6, was associated with diminished muscle protein content. These findings link the molecular regulation of mRNA translation initiation and, possibly MPS, with a measure of long-term muscle protein status in response to underfeeding. Contrary to our hypothesis, the high protein diet, fed as a percentage of total calorie intake, did not attenuate declines in mTORC1 signaling and muscle protein content compared to standard protein intake. Furthermore, the inhibition of mTORC1 associated protein translation during CR did not appear to be regulated by miR. Overall, findings from this study indicate that prolonged CR alters protein translation and downregulates mTORC1 activity, independent of associated miR. Moreover, CR appears to override the protein synthetic stimulus of increased dietary protein intake, resulting in diminished muscle protein content.

The degree to which CR alters protein metabolism appears to be dependent on the magnitude and duration of the restriction (Pasiakos et al., 2015). In short-term human studies, fasting and postprandial MPS rates are downregulated within 5–10 days of 40% CR (Pasiakos et al., 2010; Areta et al., 2014; Hector et al., 2015; Murphy et al., 2015). Consuming a high protein diet, particularly high-quality protein-containing meals, attenuates these declines and restores fasting and postprandial MPS rates to levels observed during energy balance (Pasiakos et al., 2013; Areta et al., 2014). However, these effects of short-term CR and dietary protein manipulations on MPS were not reflected in concurrent changes in mTORC1 signaling. Interestingly, Miller et al. (2013) observed a tissue specific decline in mTORC1 signaling, as rpS6 phosphorylation and total protein were lower in both heart and liver, but not skeletal muscle after 6-weeks of CR (40% total energy requirements). In the present 16-week study, we observed that a 40% CR downregulated skeletal mTORC1 protein translation and phosphorylation, regardless of dietary protein intake. These findings suggest that a time-dependent adaptation to the mTORC1 signaling pathway in response to underfeeding. More specifically, short-term CR may induce (5–10 days, 40% restriction) reductions in MPS, to conserve energy and substrate availability, but this does not elicit concomitant adaptations in mTORC1. Under these circumstances, it appears that consuming higher levels of protein may saturate basal energy and whole-body protein requirements, and provide additional substrate to maintain MPS (Pasiakos et al., 2013). However, as the duration of CR is extended, the anabolic potential of skeletal muscle (and other tissues) appears to diminish. This inhibition of mTORC1 signaling and maintenance of the translation of AMPKα and PGC-1α, key regulators of energy utilization, is in agreement with Miller et al. (2012, 2013) who suggested that selective translation of key proteins during CR maintains pathways required to preserve cellular function at the expense of anabolic processes.

Estimates of muscle protein content were lower for CR than AL, and positively associated with mTORC1 signaling. Downregulated expression of rpS6, the downstream target of mTORC1 that triggers translation initiation and cellular growth (Magnuson et al., 2012), explained 36% of the variability in muscle protein content after the 16-week intervention, with lower total rpS6 indicative of lower muscle protein content. AL and CR fed rats both gained body weight during the intervention, albeit the rate at which the CR rats grew was attenuated (Gaffney-Stomberg et al., 2014). Interestingly, the extra total mass gained for the AL rats was nearly all body fat, as fat-free mass by DXA was statistically similar between AL and CR. However, CR fed rats accrued 38% less muscle protein than AL fed rats during the 16-week intervention, suggesting DXA may not be the best tool for estimating muscle protein mass in this model. We also recognize that not examining muscle mass or cross-sectional area could be considered a limitation, unfortunately at the time of sacrifice the gastrocnemius was not weighed nor were the tissue samples fixed for histology at the time of collection. Muscle mass and cross-sectional area may have provided additional information regarding the role of altered mTORC1 signaling on muscle protein content at the level of skeletal muscle during prolonged CR. Furthermore, having mixed gastrocnemius muscle mass from 12-week old Sprague Dawley rats would have been beneficial to use a baseline values and calculate difference in skeletal muscle mass following the 16-week feeding intervention. Regardless, the observation that mTORC1 signaling was highly correlated with muscle protein content, suggests that mTORC1 responses to underfeeding are largely responsible for diminished protein content.

Increased extracellular amino acid concentrations, particularly leucine, upregulates amino acid transporter expression and uptake of amino acids into the intracellular amino acid pool and produces a robust increase in mTORC1 activity (Drummond et al., 2010; Churchward-Venne et al., 2014). The high protein diets in this study expanded the extracellular branched-chain amino acid pools, but failed to modulate the expression and activity of the mTORC1 pathway. We suspect that *ad libitum* access to feed contributed to this null effect. Extracellular leucine concentrations must surpass a certain level to optimally increase cellular uptake and stimulate mTORC1 (Moore et al., 2009; Norton et al., 2009; West et al., 2011). Providing free access to feed diminished the likelihood that protein consumed at any one point during the day surpassed the leucine threshold (Norton et al., 2009). Consistent with this explanation, branched-chain amino acid levels for the high protein CR fed rats were lower than AL 10% PRO fed rats. The lower circulating BCAA were observed despite the fact that CR 32% PRO fed rats consumed twice (4.9 g·d^−1^) the amount of dietary protein per day than AL 10% PRO (2.4 g·d^−1^). Furthermore, that no physiologically relevant (≥two-fold) increase in expression of the amino acid transporters Slc38a2 and Slc7a5, with concomitant CR-induced reductions in the expression of amino acid sensing genes Map4k3 and Lars supports the theory that extracellular leucine availability was never optimal. Protein may have been stimulatory, despite CR, if food was provided at discrete times and had sufficient protein to adequately increase plasma amino acid levels.

To the best of our knowledge this is the first investigation to assess the role of skeletal muscle miR expression on mTORC1 signaling following adaptations to prolonged CR. Recent studies have indicated miR expression play a role in the regulation of muscle mass (McCarthy and Esser, 2007), via the mTORC1 pathway (Jin et al., 2013; Wei et al., 2013; Jia et al., 2014; Zacharewicz et al., 2014). In contrast, miR in the current investigation were not impacted by CR and had no effect on mTORC1. Using a targeted assessment of specific miR in the current study may have limited our ability to capture altered expression other potentially relevant miR that could have been measured using a microarray or miR-seq. However, a global approach was not taken because our intent was to determine if miR previously shown to regulate specific mTORC1 associated proteins were also influenced by CR or high protein intake. Findings from the present investigation indicate that alterations of mTORC1 signaling following sustained CR are not regulated by the expression of miR-99a or miR-100-5p.

In conclusion, data from this study provide a link between mTORC1 signaling, muscle protein content, and prolonged underfeeding. The systematic downregulation of mTORC1 signaling activity with CR, regardless of protein intake, was likely the result of inhibited protein translation and subsequently lower phosphorylation of Akt, mTOR, p70S6K, and rpS6. Furthermore, while the effects of CR on mTORC1 were clearly demonstrated, they appear to be independent of miR expression. These data also demonstrate that high dietary protein intake alone, when provided as a percentage of total energy and evenly distributed in food consumed *ad libitum*, is not sufficient to attenuate mTORC1 adaptations to prolonged CR.

## Author contributions

JM and SP: Study design, oversight, and sample collection. LM, DR, MB, YE: Completed experiments and analyzed and managed data. LM, DR, AY, JM, RF, SP: Data interpretation and manuscript preparation. Reviewed by all authors.

## Funding

This material is based on the work supported by the US Army Medical Research and Material Command, the Dairy Research Institute, and the USDA under agreement No. 58-1950-4-003. LM was supported by the T32 NIDDK training grant # 5T32DK062032-23 and DR was supported by the NIA K01 award # KAG047247A-A1.

### Conflict of interest statement

The authors declare that the research was conducted in the absence of any commercial or financial relationships that could be construed as a potential conflict of interest.

## Abbreviations

Akt: protein kinase B
AL: *ad libitum*
AMPKα: AMP-activated protein kinase α
B2M: Beta-2-Microglobulin
CR: calorie restriction
DXA: dual energy X-ray absorptiometry
GAPDH: glyceraldehyde-3 phosphate dehydrogenase
Lars: Leucyl-tRNA Synthetase
Map4k3: Mitogen-Activated Protein Kinase Kinase Kinase Kinase 3
miR: microRNA
MPS: muscle protein synthesis
mTORC1: mechanistic target of rapamycin complex 1
PGC-1α, Peroxisome proliferator-activated receptor gamma, coactivator 1: alpha
Ppara: Peroxisome proliferator-activated receptor alpha
Pparg: Peroxisome proliferator-activated receptor gamma
PRO: protein
p70S6K: 70 kDa S6 kinase
RDA: recommended dietary allowance
rpS6: ribosomal protein S6
Sirt1: Sirtuin 1
Slc38a2, Solute Carrier Family 38: Member 2
Slc7a5, Solute Carrier Family 7, Amino Acid Transporter Light Chain: L System
U6, RNA: U6 Small Nuclear 1.
