# Supplementary material for: Prolonged Calorie Restriction Downregulates Skeletal Muscle mTORC1 Signaling Independent of Dietary Protein Intake and Associated microRNA Expression
Source: Front Physiol. 2016 Oct 5;7:445. doi: 10.3389/fphys.2016.00445 (PMC5050214; doi:10.3389/fphys.2016.00445)
Supplement: Supplementary file 1 [file Table1.DOCX]

Supplementary Material

**Prolonged calorie restriction downregulates skeletal muscle mTORC1 signaling independent of dietary protein intake and associated microRNA expression**

Lee M. Margolis, Donato A. Rivas, Maria Berrone, Yassine Ezzyat, Andrew J. Young, James P. McClung, Roger A. Fielding, Stefan M. Pasiakos^*^

*** Correspondence:** Stefan M. Pasiakos : [stefan.m.pasiakos.civ@mail.mil](mailto:stefan.m.pasiakos.civ@mail.mil)

**Supplemental Table 1.** mRNA and miR Targets and Function

| **Name** | **Function** |
| --- | --- |
| ***mRNA*** |  |
| Slc38a2 | Transporter; sodium dependent cellular uptake of amino acid |
| Slc7a5 | Transporter; cellular uptake of large neutral amino acids |
| Map4k3 | Amino acid sensor stimulating activation of p70S6K |
| Lars | Leucine sensor interacting directly with Rag GTPase to active mTORC1 |
| Sirt1 | NAD-dependent protein deacetylase contributing to regulation of energy utilization |
| Ppargc1a | Transcription factor regulating genes involved in mitochondrial biogenesis, lipid metabolism and carbohydrate metabolism |
| Tfam | Mitochondrial transcription factor, promotes mitochondrial biogenesis |
| Ppara | Transcription factor regulating fatty acid uptake and oxidation |
| Pparg | Transcription factor regulating fatty acid storage |
| ***miR*** |  |
| miR-16-5p | Inhibits IGF-1R and regulates cell proliferations |
| miR-99a-5p | Inhibits gene expression and protein translation of mTOR |
| miR-100-5p | Inhibits gene expression and protein translation of mTOR and Raptor |
| miR-128a3p | Inhibits phosphorylation of Akt |
| miR-133a-3p | Inhibits protein expression of IGF-1R and phosphorylation of Akt |
| miR-199a-3p | Inhibits gene expression of mTOR |
| miR-221-3p | Stimulates protein translation of Akt through inhibition of PTEN |

Slc38a2: Solute Carrier Family 38, Member 2; Slc7a5: Solute Carrier Family 7, Amino Acid Transporter Light Chain, L System; Map4k3: Mitogen-Activated Protein Kinase Kinase Kinase Kinase 3; Lars: Leucyl-tRNA Synthetase; Sirt1: Sirtuin 1; Ppargc1a: Peroxisome proliferator-activated receptor gamma coactivator 1-alpha; Tfam: mitochondria transcription factor; Ppara: Peroxisome proliferator-activated receptor alpha; Pparg: Peroxisome proliferator-activated receptor gamma.
